# Mr. Bennion, on the Gibraltar Fever

**Published:** 1805-08-01

**Authors:** James M'Grigor

**Affiliations:** Windsor


### To Dr. BATTY.

Sir,

THE enclosed letter from Thomas Bennion, Esq. Garrison Surgeon of Gibraltar, I received two days ago. We have had but very scanty accounts of the disease which so fatally raged there; this account therefore, though imperfect, may perhaps be acceptable. An account from Mr. Bennion must be particularly interesting, as he was surgeon of the 10th Regiment while the disease raged, and it will be recollected that the mortality in that regiment was so very much smaller than in any other corps in the garrison. See the account published in the Medical and Physical Journal, by the Ordnance Surgeon at Gibraltar.

I am, &c.

*Windsor, July* 4, 1805. JAMES M'GRIGOR.

### To Mr. M'GRIGOR.

Sir,

The Garrison is now perfectly clear of that cruel disease which proved so fatal both to the soldiers and to the inhabitants here. You ask me, what the disease is, or to what class it belongs? I have never been in the West Indies, or on the continent of America, I consequently cannot positively say that it was the yellow fever; but it appeared to me, to be that very disease as described by authors, particularly by Dr. Chisholm. It likewise often appeared in the shape of a disease which you and I saw often in Egypt, the plague; but I shall give you some description of it, and will divide the symptoms as they appeared, into two stages.

In the first, the patient is seized without any previous notice, with giddiness, pain of the head, slight sickness at stomach, darting pains from the head to the back, and spasmodic affection of the calves of the legs. Sometimes there were rigors, and in several cases the sickness at the stomach did not come on for some hours after the first attack. The breathing was very hot, there was incessant sighing, the greatest dejection of spirits, with apprehensions of the most distressing nature. The patient said he felt as if severely beaten; the fear of death is very great, and friendship for the nearest kin is extinguished. The tongue was at the beginning very white; a bad taste was complained of; the sense of smelling was imperfect or depraved; the visage was extremely distressed; there was an unwillingness to speak, a desire to remain in a horizontal posture, and great tottering on any attempt at motion.

The countenance on the first attack became suddenly sallow; in the course of a very short time however it became red, full, and bloated, with the exact appearances of intoxication. Drowsiness and sleep followed in a few hours, when a little moisture came out on the skin; this appearance at this stage was however delusive, and often deceived the practitioner. It suddenly left the patient, and was succeeded by the most intense heat, that gave a smarting sensation to the fingers when applied to the skin. There was at this time a most uncommon and offensive smell from the whole body. The eyes were now much inflamed, there was violent pain in the temples and over the arches of the eye-brows, darting to the orbits.

The pulse from first to last was greatly increased in quickness; in some it was rather hard, though in more it was small and soft, and never so strong and firm as in inflammatory diseases.

The thirst was less than it is generally in acute diseases. There was a strong pulsation of the carotid arteries, and an evident enlargement of the jugular veins.

The colour of the skin at first approximated to that of the lilac, cocklicoque, violet or poppy, and changed as the disease advanced to a deep dark yellow. By the administration early of strong emetics and purgatives on the first attack, the yellowness seldom appeared, and every other bad symptom was averted.

When these had not been exhibited; and in some cases, where the disease from first appeared in a more aggravated form, the second set of symptoms, or what I called the secondary, soon appeared; the patient was very comatose, there was much tremor of the limbs, frequently an incessant vomiting of a black matter, with a convulsive hiccup. The eyes were drawn in a direction alternately from the nose to the temples in the most frightful manner, with nearly a total blindness.

The skin was now parched up with a burning heat, or covered with a clammy offensive sweat.

The body was covered with petechiæ and vibices, swellings appeared under the arm-pits and groins, which frequently degenerated into large abscesses. Foul gangrenous sores appeared on the back, and carbuncles were seen on different parts of the body. There were hæmorrhages from the nose, ears, mouth, and pores of the body, with every appearance of a total dissolution of the blood-vessels. Then the fæces and urine were passed involuntarily, and the other usual symptoms indicated speedy dissolution.

As to the nature of this disease we were by no means agreed; different medical councils were held in the garrison, and at first it appeared to be the opinion of a majority that the disease was not contagious; a fatal opinion to be acted on, as subsequently appeared.

I arrived here a few days after the disease made its appearance, and of course, could not give any opinion about it, until I had thoroughly seen the disease and enquired into its history. General Barnett again strongly urged us to say whether it was contagious; alas! I soon discovered it to be the nearest relation to the plague; some cases from first putting on the appearance of this disease, others of the typhus gravior, and others of the yellow fever.

As to the mode of treatment, that which I followed is very simple, and is soon described. I have little to remark on on the plans of others; it is known that they were not equally successful. Some of our ablest men were inclined to interrupt the course of the disease but little, and gave little but diluents and cordials. Others bled very freely; and others gave the bark very liberally and early.

My mode of treatment was as follows. My first step was invariably to put every patient into a bath, which was generally warm; when taken out, the body was well rubbed with soaped flannel, and then he was put into bed. If the powers of life were strong, an emetic solution was next given, of Tartar emetic and Glauber salts. This solution generally operated pretty smartly both on the stomach and bowels; and when it did its office well, I have frequently had little more to do but to remove debility, the patients being often well on the third day. If the solution, persevered in for some time, did not operate, which was frequently the case at first, the stomach and bowels being very insensible, I gave calomel, and continued giving it either by itself or with jalap, or with the compound extract of colocynth. I endeavoured by all means to keep up the alvine discharge; when obtained, the patient was found perfectly relieved, and free from fever. If not, the fourth or fifth day generally put an end to all enquiry. But in many, no evacuation could be procured by any means; in others there was a violent diarrhœa.

I dissected the bodies of a few; the general appearances were, the destruction of the internal coat of the stomach, inflammation and ulceration of the intestines, sometimes of other of the abdominal viscera.

I have little more to say, than that after procuring evacuations, I prescribed saline medicines when little fever remained; but when the disease continued after the third day, it frequently turned out to be the severest typhus. I then found the greatest benefit by Dr. Carmichael Smyth's mode of treatment, as laid down in his book on the Winchester fever. Opium or bark did not succeed; when liberally given, I perceived them evidently doing mischief. The ship is getting under way, and I must conclude.

Your's, &c.

THOMAS BENNION.

*Gibraltar, April* 8, 1805.

